# Handheld mechanical nociceptive threshold testing in dairy cows – intra-individual variation, inter-observer agreement and variation over time

**DOI:** 10.1111/vaa.12159

**Published:** 2014-04-16

**Authors:** Peter M Raundal, Pia H Andersen, Nils Toft, Björn Forkman, Lene Munksgaard, Mette S Herskin

**Affiliations:** * Knowledge Centre for Agriculture, CattleAarhus N, Denmark; † Department of Large Animal Science, University of CopenhagenCopenhagen, Denmark; ‡ Department of Clinical Sciences, Swedish University of Agricultural SciencesUppsala, Sweden; § National Veterinary Institute, Technical University of DenmarkKongens Lyngby, Denmark; ¶ Department of Animal Science, Aarhus University, AU-FoulumTjele, Denmark

**Keywords:** dairy cows, mechanical nociceptive threshold, pain

## Abstract

**Objective:**

To examine the use of handheld methodology to assess mechanical nociceptive threshold (MNT) on cows kept loose-housed.

**Study design:**

Prospective randomized partial cross-over experimental study. A one-factor (test day) design was used to evaluate MNT over time.

**Animals:**

One hundred and fifteen healthy, loose-housed Danish Holstein cattle.

**Methods:**

We evaluated intra-individual variation, inter-observer agreement and variation over time of MNT using two handheld devices and two stimulation sites. Mechanical, ramped stimulations were performed with an algometer (6.5 mm diameter steel probe, 0–10.0 kgf) or an electronic von Frey device (plastic tip with diameter 0.8 mm, 0–1000 gf). Each cow received 5–6 consecutive stimulations within a 2 × 5 cm skin area on the dorsal or lateral aspect of the left third metatarsus until an avoidance reaction occurred. We investigated the difference in precision [expressed as coefficient of variation (CV)] between the combinations of devices and stimulation sites. The inter-observer agreement and the difference in MNT between test day 1, 3, 7, 10 and 24 were investigated for selected combinations. Data were analysed in mixed models and Bland-Altman as relevant.

**Results:**

The CVs did not differ [range 0.34–0.52 (*p *=* *0.1)]. Difference between observers (95% limits) was 0.2 kgf (2.8) and 4 gf (369) for the algometer and von Frey device, respectively. Mechanical nociceptive threshold increased from 361 on test day one to 495 gf on test day 24 (*p *<* *0.01).

**Conclusion and clinical relevance:**

All methods showed a high degree of intra-individual variation, and no combination of device and stimulation site showed superior precision. Mean difference between observers was low, and MNT was not consistent over time. Further development of the methods is required before they can be used in research to investigate possible relations between claw lesions and hyperalgesia.

## Introduction

Claw disorders and lameness are considered to be among the major welfare problems in intensive milk production (Anonymous 2009). Claw lesions often are associated with pain (O'Callaghan et al. 2003; Dyer et al. 2007) and persistent pain may lead to hyperalgesic states via peripheral and central sensitisation of the nervous system (Anderson & Muir 2005). Nociceptive threshold testing can be used to investigate hyperalgesia associated with clinical conditions (Love et al. 2011). In previous studies in dairy cattle, mechanical nociceptive thresholds (MNT) have been used to quantify hyperalgesia associated with claw disorders. Mechanical nociceptive stimulation has been applied to the skin of the dorsal part of the metatarsus/metacarpus by use of a blunt pin, driven by a pneumatic actuator and attached to the leg with a cuff (Whay et al. 1997, 1998; Laven et al. 2003). However, this method requires handling and restraint of the cows, potential stressors which might influence the nociceptive thresholds (Rushen et al. 1999; Herskin et al. 2004, 2007). Furthermore, in the modern dairy industry, many dairy cows are kept in loose-housing systems. Handheld devices for MNT testing have been used in other animal species (horses: van Loon et al. 2012; dogs: Pieper et al. 2011; sheep: Stubsjøen et al. 2010 and pigs: Di Giminiani et al. 2013). Thus, in order to be able to quantify MNT in modern dairy production, handheld methods, which can be used on freely behaving dairy cows kept in their home environment, seem to offer a good alternative but such method have not yet been investigated.

Application of mechanical force on the skin creates a pressure which spreads into the skin and underlying tissue. The pressure causes deformation that may lead to activation of nociceptors in different layers of the tissue depending on the size and shape of the probe (Treede et al. 2002). To reduce the spread of pressure the amount of distensible tissue underlying the stimulation site should be minimized (Love et al. 2011). The dorsal aspect of the metatarsus has been used to investigate hyperalgesia related to bovine claw disorders (Whay et al. 1997, 1998; Laven et al. 2003), but other anatomical locations have not yet been investigated.

A prerequisite for the study of changes in the pain processing system over time or in response to an intervention is that threshold quantification remains stable over time (Potter et al. 2006). This has been evaluated in other species using handheld equipment, e.g. in humans (Jensen et al. 1986; Potter et al. 2006), horses (Haussler & Erb 2006), pigs (Janczak et al. 2012) and sheep (Stubsjøen et al. 2010) but has not been reported in cattle.

For a handheld algometer to be used in the clinic or in large scale studies, the dependency of observer must be known. This has been investigated in human subjects, where no bias and a reliable inter observer correlation was found between five observers (Chesterton et al. 2007). In dairy cows, inter-observer dependency has not yet been reported.

Thus, as part of the initial work to be able to assess changes in MNT associated with claw disorders in dairy cows, the aim of the present study was to develop handheld methodology appropriate for this purpose. Firstly, we aimed to investigate intra-individual variability of MNT on dairy cows kept in their home environment and relate this to type of the mechanical pressure device and anatomical site of stimulation. Secondly, we aimed to investigate inter-observer agreement between two observers using both devices and the dorsal stimulation site. As a third aim, we investigated the variation over time using the electronic von Frey and the dorsal stimulation site. Finally, *post hoc,* we evaluated the effect of the experimental cow's behavioural response to the initial presence of the observer on the subsequent MNT.

## Material and methods

### Ethical statement

The procedures and housing of the animals complied with the criteria given by the Danish Animal Experiments Inspectorate as procedures that do not require specific approval.

### Animals and housing

Both experiments were carried out at the Cattle Research Centre, Tjele, Denmark between April and July 2011. The 140 cows in the resident herd were kept in two groups in a loose-housing system with resting areas in cubicles (120 × 225 cm with mattresses and limited sawdust) and slatted floors. The cows had 24 hour access to a Total Mixed Ration in individual computer operated feeding boxes (Roughage Intake Control (RIC); Insentec B.V, The Netherlands) and to one milking robot per group (VMS, De Leval A/S, Denmark). During Experiment 2, all cows were claw trimmed at day 8 or 9 as part of the normal management of the herd.

### Study design

#### Experiment 1 (Exp. 1)

Thirty-five cows were included in a 2 × 2 factorial study with type of device: algometer (A) *versus* (vs.) electronic von Frey (vF) and stimulation site: dorsal (D) *versus* lateral (L) aspect of the left metatarsus as the two factors, resulting in four combinations (AD, AL, vFD and vFL). Thirty-five combination sequences were listed in a balanced orthogonal Latin square cross-over design (Jones & Kenward 2003) with one combination per day on four consecutive days. The experimental cows were allocated to the list of sequences by a random integer generator (www.random.org). Eight cows were excluded *post hoc* (seven had a lameness score >2 *post hoc*, one cow left the cubicle before stimulation), thus in total, 21, 22, 17 and 23 cows received combinations AD, AL, vFD and vFL, respectively.

#### Experiment 2 (Exp. 2)

Ninety cows were included and blocked by parity, lactation stage, days to expected calving and milk yield (based on a 6-day average, 14–20 days prior to the experiment) in two blocks. Ten cows (3, 4 and 3 cows of parity 1, 2 and 2+, respectively) were allocated as reserve cows. The two blocks were assigned to either the A or vF device by a computer coin flipper (www.random.org). Three plus three single nociceptive stimulations were appointed to each cow on experimental day one and three in an observer depended (either first observer P, then observer K or reverse) and random cross-over sequence. Furthermore, cows stimulated with vF were retested at day 7, 10 and 24 by observer P. Ten reserve cows replaced ten cows which left the cubicle before testing at first test day. Sixteen cows were *post hoc* excluded; three cows were not found in cubicles at any test day, and 13 were excluded due to *post hoc* recorded lameness. In total, 64 cows, 31 with A and 33 with vF, were tested.

### Criteria for inclusion and exclusion

Criteria for experimental inclusion were: lactating Danish Holstein, more than 30 days in milk (DIM), more than 60 days before expected calving, lameness score below three within 4 weeks before the experiment [performed by trained technicians using the scoring system described by Thomsen et al. (2008)] and milk somatic cell count below 450.000 within 3 weeks before the experiment. The applied criteria for experimental exclusion were cows that: could not be found in cubicle on test days, left the cubicle before first stimulation, were not clinically healthy based on visual inspection by trained veterinarian combined with rectal temperature outside the interval between 38.0 and 39.0 °C, had signs of oestrus on test days, kicked during testing or had a lameness score of more than two within 2 weeks after the experiment. The first ten cows that were excluded on the first test day in Exp. 2 were replaced by reserve cows.

### Nociceptive testing

We used two mechanical devices. A Wagner Pain Test Algometer (FPK 20; Wagner Instruments, CT, USA) with a 6.5 mm diameter flat steel probe with rounded edge, hence a stimulation area of approximately 33 mm^2^, and an Electronic von Frey Anesthesiometer (IICT Inc., CA, USA) with a 1000 g force rigid plastic tip (pipette) attached. The tip was hollow with an outer and inner diameter of 0.8 and 0.5 mm given a ring shaped surface with an area of approximately 0.3 mm^2^ and with sharp contour of the inner and outer edges. Range of measurements was 0–10.0 kg force (kgf) and 0–1000 g force (gf) for the algometer and the von Frey device, respectively. The maximum ranges were used as safety end points to avoid tissue damage. Stimulation sites were 2 × 5 cm areas either along the middle third of the dorsal aspect of the left caudal cannon bone or along the middle third of the lateral aspect of the left cannon just dorsal to the deep flexor tendon. Threshold testing was performed between 9.45 and 15.30 hours. In Exp. 2, observer K was given a few minutes of instruction and practice on non-experimental cows just prior to the experiment. The observer(s) wore blue overalls similar to other visitors in the barn. When an experimental cow was identified in a cubicle, the observer approached the cow's left hind leg until a distance of approximately 50 cm. At this initial presence, the observer stood still for approximately 15 seconds and ensured, by eye contact, that the cow was notified. If a cow was lying down, she was gently encouraged to get up. The cow behaviour with the highest rating (Table1) during the initial presence of the observer was recorded as a ‘first presence’ response. The observer(s) then performed 5 (Exp. 1) or 6 (Exp. 2) consecutive stimulations. Within the stimulation site, the cows were stimulated on different spots, approximately 1 cm apart and with an approximately 30 seconds' inter-stimulation interval. Shifting of observer in Exp. 2 was done quietly between the third and fourth stimulation. Each stimulus was performed as a ramped pressure, until the cow expressed a behavioural avoidance reaction that removed the leg from the probe or until the safety end point was reached, both of which terminated the stimulus and was recorded as the ‘avoidance’ response (Table1). The pressure applied at this point, given in kgf (A) or gf (vF), was recorded as the MNT value. The cow behaviour with the highest number (Table1) was recorded as the ‘inter-stimulus interval’ response. The response was recorded after each stimulation by the performing observer except on test day one and three in Exp. 2, where the non-performing observer did the recordings. All stimulations were performed when the experimental cows were standing in the cubicle with approximately parallel legs and even weight distribution (assessed by visual inspection). No restraining procedure was done except the observer's positioning near the cows' left hind leg, and cows were free to express any movements including leaving the cubicle.

**Table 1:** Ethogram describing the types of behaviour recorded during the tests of MNT on unrestrained loose-housed dairy cows. Behaviour was recorded as the ‘first presence’ response (the response when the observer approached the cow and stood still close to the left hind leg before initiating the test procedure), the ‘avoidance’ response and the ‘inter-stimulus interval’ response. For the ‘first presence’ and the ‘inter-stimulus interval’ response, only the behaviour with the highest rating number was recorded. The rating numbers are not on an ordinal scale

Rating no.	Behaviour	Description
1	Not orientated at observer	Head forward or bended sideways <90° relative to straight forward. No stepping
2	Orientated at observer	Head held sideways towards observer more than 90° relative to straight forward. No stepping
3	Head high	Head held min. 20 cm above the dorsal line
4	Minor stepping	Stepping <5 times and hind feet lifted max. 5 cm
5	Major stepping	Stepping more than five times and hind feet lifted more than 5 cm
6	Leg lift	Lifting the foot more than to the level of the fetlock
7	Quick leg lift	Quickly lifting leg
8	Kick	Cow kicked at observer
9	Moving	Moving the rear end away from the test person
10	Leaving	Leaves the cubicle

### Variables and data analysis

Outcome variable was the MNT values, related to the force applied at each avoidance response. The MNT was measured in the units given by the devices: kgf (algometer) or gf (von Frey) and can be converted to SI units by 1 Newton (N) equals 9.81 kgf. In the absence of an avoidance response, at safety end points, the maximum threshold value was assigned and included in the dataset.

One intra-individual coefficient of variation (CV) was calculated per cow per combination in Exp. 1 as the standard deviation, divided by the mean of the obtained measurements and used as an indicator of precision of each of the four combinations. In sessions where a cow left the cubicle after the first stimulation (i.e. only one measurement per cow), the CV could not be calculated and were hence not included in the analysis of CV. Comparison of CVs between combinations of device and stimulation site was carried out by linear mixed model with combination as fixed and cowID as random effects. Results are presented as estimated means and standards errors derived from the model. Tukey contrasts were used for pairwise comparison.

Inter-observer agreement was assessed by the Bland-Altman method (Bland & Altman 1986), where the mean MNT was calculated per observer and cow. The differences between the observer means for each cow were plotted against one mean per cow, calculated from the means per observer per cow. The overall mean difference between the observers was used as an estimate of the bias of one observer relative to the other. Limits of agreement were calculated as the 95% confidence limits of the overall mean (±1.96 × SD). A standardised agreement index (AI), given as AI = 1 – (2SD_mean-difference_/mean level), was calculated for each probe type. A positive AI supported agreement and values larger than 0.5 indicated good agreement (Kampen et al. 2004).

To evaluate the effect of the behavioural response during the initial presence of the observer on the subsequent MNT, cows with a recorded behaviour rating more than two at the ‘first presence’ response were categorized as ‘fearful’. This effect could only be evaluated in Exp. 2, as all eight ‘fearful’ cows in Exp. 1 left the cubicle before any stimulation.

Effects of experimental day, stimulation site and behavioural response to the initial presence of observer were analysed by generalised linear mixed models. Initial models were analysed for explainable power by using Akaike's Information Criterion and subsequent evaluated by residual plots. Fixed effect in Exp. 1 was stimulation site and random effects were cow, stimulation number and experimental day. Fixed effects in Exp. 2 were behavioural response to the initial presence of the observer and experimental day. Random effects were cow and stimulation number. Tukey contrasts were used for comparison and significance accepted for *p* < 0.05. Results are presented as estimated means and standard errors derived from the statistical models. Statistical analysis was done using R (R Development Core Team 2011).

## Results

**Figure 1:**
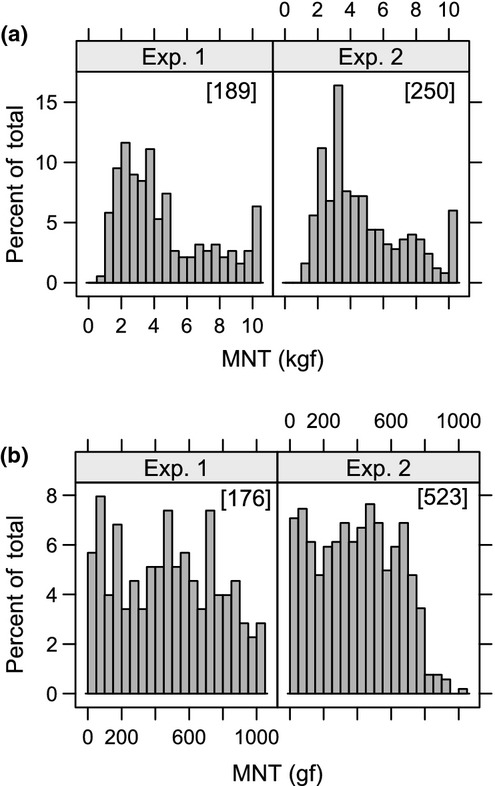
Distribution of measured MNTs with algometer (a) and von Frey (b) in Exp. 1 and 2 involving loose-housed dairy cows. The total number of measurements is given in [ ]. The percentages of censored observations are shown as the four right columns.

Out of 700 planned stimulations, 365 (189 A and 176 vF) resulted in MNT measurements in Exp. 1 (Fig.1, left side).

This level of drop-outs was due to the fact that the cows – at any time during testing – were free to leave the cubicle. Hence, relative to the number of cows that accepted the first presence of the observer without leaving the cubicle, 80, 75, 70, 67 and 63% of the cows accepted the one to five following stimulations, respectively. No major differences in the level of successful stimulations were found between combinations. This resulted in 80 complete records (all five stimulations performed per cow and combination) and 6, 3, 7, and 7 incomplete records with 4, 3, 2 and 1 stimulation performed, respectively.

In Exp. 2, the 1680 planned stimulations (480 with A and 1200 with vF) resulted in 773 MNT measurements (250 A and 523 vF, respectively, Fig.1, right side). Relative to the number of cows that accepted the first presence of the observer, 86, 73, 64, 61, 57 and 55% of the cows accepted the one to six following stimulations, respectively. No major differences in the level of successful stimulations were found between the order of observers or test days. There were 108 complete records (all six stimulations) and 3, 8, 6, 17 and 26 incomplete records with 5, 4, 3, 2 and 1 stimulation, respectively.

The precision, given as CV, did not differ between the four combinations, although the AD tended to have lower CV than the vFD combination (Table2). On five occasions, the CV could not be calculated as these stimulation sessions had only one successful measurement each. One stimulation session in AL was excluded as all five stimulations reached the safety end point and thus all were given the maximum value (10.0 kgf).

**Table 2:** Precision of the four combinations of device and stimulation site used to quantify MNT in unrestrained loose-housed dairy cows. Precision is given as the intra-individual coefficient of variations (CVs) based on one calculated CV of MNT per cow and presented as the estimated mean and standard errors (SE) derived from the statistical models. Differences analysed as mixed models using Tukey contrasts. *p* Values are given for pairwise comparison between combinations

CV		*p* Values		
		AD	AL	vFD
Estimated mean ± SE				
AD	0.34 ± 0.06			
AL	0.41 ± 0.05	0.8		
vFD	0.52 ± 0.06	0.1	0.4	
vFL	0.48 ± 0.05	0.2	0.7	1.0

A, algometer; vF, von Frey. D and L, dorsal and lateral stimulation site, respectively.

**Figure 2:**
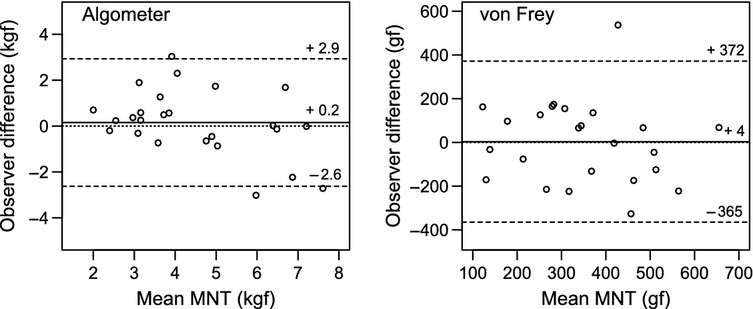
Bland-Altman plots of inter observer agreement for the algometer and von Frey device probe used to quantify MNT in unrestrained dairy cows in the home environment. The difference between observers (mean MNT per cow obtained by observer K – mean MNT per cow obtained by observer P) is plotted against the mean ([observer K + observer P]/2). Solid line: Overall mean difference between observer K and P. Dashed lines: Limits of agreement as the 95% confidence limits of the mean difference (solid lines). Dotted lines represent total average agreement.

The mean difference and limits of agreement between the observers are illustrated in Fig.2. By visual inspection, the average inter-observer differences were considered as small for both devices. However, the 95% limits of agreement composed 5.5 kgf and 737 gf out of 10.0 kgf and 1000 gf range for the A and vF devices, respectively. With the vF device, one ‘outlier’ observation (observer difference = 537 gf) had a major influence on the average inter-observer difference as well as the standard deviation of the differences. Agreement indexes were 0.58 and 0.37 for the A and vF devices, respectively.

**Figure 3:**
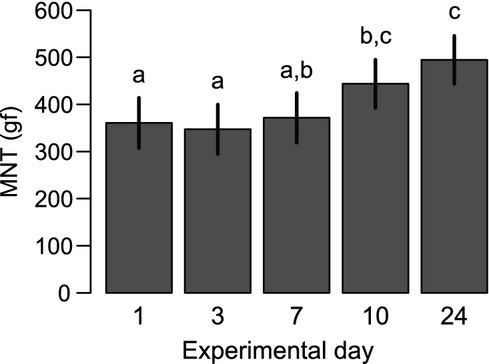
Estimated means of MNT from unrestrained dairy cows kept in the home environment of each experimental day and stimulated with the electronic von Frey device. Different superscripts indicate significant difference. Error bars represent estimates of standard errors derived from the statistical models.

**Figure 4:**
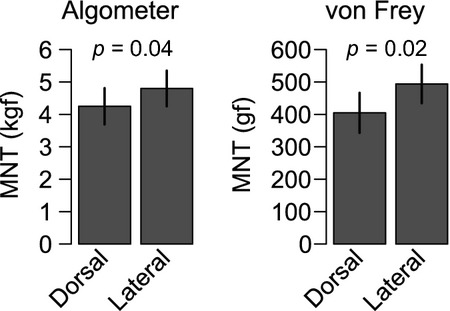
MNT quantified as estimated means on dorsal and lateral stimulation site using either algometer or von Frey device. During tests, dairy cows were unrestrained and kept in their home environment. Data analysed as mixed model and contrasts evaluated by Tukey method. Error bars represent estimates of standard errors derived from the statistical models.

Variation over time is illustrated in Fig.3. The mean MNT increased significantly during the experiment. As the mean difference between observers was low, the measurements per cow were pooled at day one and day three. By comparing the two stimulation sites, MNT was significantly lower on D than L site for both devices (Fig.4).

*Post hoc* we evaluated the effect of the behavioural response to the first presence of the observer on the MNT. For cows categorized as ‘fearful’ (recorded with a behaviour rating number higher than two at ‘first presence’), the subsequent MNT was significant lower than ‘non-fearful’ cows when tested with the A device (mean MNT ± SE: 3.91 ± 0.51 kgf *versus* 4.88 ± 0.36 kgf, *p* = 0.02). There was no difference within cows tested with the vF device.

### Adverse events

When using the A device, the application of force in the high end of the range could be a physical challenge to the observers. At the high end of the range, the probe also tended to slide off the dorsal stimulation site. Keeping the plastic tip of the vF device constantly perpendicular to the skin surface during the stimulations was a practical challenge. Moreover, the plastic tip seemed sensitive to side load forces and tended to bend at the high end of the range.

## Discussion

As part of the development of a handheld methodology appropriate for quantification of MNT testing in dairy cows kept in loose housing, we investigated the short term precision of four combinations of probe diameter and stimulation site, determined the inter-observer agreement and the temporal consistency of mean MNT over a 24 day period. The level of precision did not differ significantly between the four treatments [algometer-dorsal (AD), algometer-lateral (AL), von Frey-dorsal (vFD) and von Frey-lateral (vFL)], and the results showed good agreement between observers, however, with wide limits. Agreement Index was found to be just above ‘good’ (Kampen et al. 2004) for algometer but not for von Frey device. Over time, the estimated mean of MNT increased significantly. Taken together, the results suggest that further development is needed to be able to increase the reliability of the handheld methods, if they should be applicable to quantify bovine mechanical nociceptive threshold (MNT) on freely behaving dairy cows kept in their home pens.

When the precisions of the four combinations of devices and stimulation sites were compared, our results indicate that even though the AD combination tended to have lower coefficient of variation (CV) than the vFD combination, no combination was superior to others quantified as having a lower intra-individual CV. In studies involving healthy humans, using a variety of methodologies, the CV ranged between 10 and 48% (Cathcart & Pritchard 2006; Ylinen et al. 2007). Our results are the first reported to address the intra-individual CV in MNT testing in cattle and lie within the range found in human subjects, although close to the upper limit. In a study with dairy calves, where laser stimulation was used to test the thermal nociceptive threshold and applied consistently and without an observer being close to the animal, the intra-individual CV was reported as 36% (Veissier et al. 2000).

Our relatively high range of CVs could be due to several causes. A first source of variation could be that cows responded to various degrees of different sensations: touch, pain detection or pain tolerance even within the same stimulation session. Hence low MNTs might be due to activation of mechanoreceptors by touch but the exact frequency cannot be indicated as we could not control whether only the mechanoreceptors or mechano- and nociceptors were activated. Recordings of high values, including the censored safety end points, might have been due to lack of activation of the nociceptors or cows may have responded at the pain tolerance threshold. In addition, they could have been affected by a degree of stress induced hypoalgesia which can occur in cattle after exposure to acute stressors (Herskin et al. 2004). The cow's unfamiliarity with the observer(s) and the testing procedure in our study could have been potential stressors. To reduce variation in pressure pain threshold testing in humans, test persons are instructed to which threshold they should respond to (Potter et al. 2006) and in sheep a pre-test habituation procedure has been suggested to reduce the variation in the response to the stimulus (Stubsjøen et al. 2010). As a second source of variation, increased probe diameters have been shown to be related to larger variation in mechanical nociceptive thresholds (Taylor & Dixon 2012b), this factor could explain some of the variation with the algometer. A third possible source of error could be the influence of environmental stimuli other than the observer. To avoid stress due to novel surroundings, the experimental cows in the present experiment were kept in their home environment, where environmental stimuli could not be fully controlled. A fourth source of variation was the low level of control with the rates of the ramped stimulations as otherwise recommended for mechanical nociceptive testing (Jensen et al. 1986; Leuchtweis et al. 2010). Hence the rate could have differed between stimulations resulting in increased variation. Further, although the latency to respond was not measured in our study, the cows generally responded to the ramped stimuli within seconds, according to the observers. If so, this results in a relatively high rate compared to another MNT study in cattle, where the threshold was reached in an average of 66 seconds. (MNT: 13.3 N, rate: 4.5 N/seconds) (Whay et al. 1998). A high rate may jeopardize the precision of the measured MNT values as relatively small variations in response time (subjects or observers) would result in relative large variations in MNT. Moreover, given the high rate and the high force of the algometer, the stimulated leg could have been pushed away, resulting in a false response.

In summary, to reduce the variation in MNT testing in dairy cows in future studies, we suggest to use a pre-test habituation procedure, relative small probe sizes, a controlled environment and stimulations devices where the rate can be controlled.

This study involved two observers. For both devices, the mean inter-observer difference was low. However, only the algometer showed a good agreement index. The low level of agreement between the observers using the von Frey device may have resulted from the one ‘outlier’ observation. This observation came from one cow where only one measurement per observer was obtained as the cow left the cubicle after the first stimulation on both test days. The limited number of successful stimulations (2/12) reduces the reliability of this data point. We decided not to exclude this observation due to lack of predefined exclusion criterion, and given that 34 out of 57 cows in our study also had <12 successful stimulations. This emphasises the challenges with no restraint of experimental cows in our study. However, our results with the algometer suggest that it is possible to obtain good inter-observer agreement even when the observers were unfamiliar to the animals.

We found a significant increment in MNT over 4 weeks. A slightly, but significantly, increase in pain pressure threshold over 5 weeks has also been found in an algometry study in healthy humans (Jensen et al. 1986). The authors suggested an effect of reduced anxiety of the subjects as they were getting familiar to the procedure. This could be supported by our finding where cows that scored ‘fearful’ towards the human presence but accepted stimulations, had lower threshold values than less fearful cows when stimulated with the algometer. Another reason for the increase in MNT over time could be due to an increased familiarisation to being touched, since the same cows were stimulated repeatedly during the experimental period, which potentially could cause a habituation effect to the initial tactile phase of stimulation. A learning effect, which has been suggested to cause a decreased MNT over several weeks in horses (Chambers et al. 1990), could not be supported by our results. Whatsoever the reason, a test method should have acceptable test-retest equity to be used in examinations of interventions or changes over time (Potter et al. 2006). Therefore, our methods need to be further developed to minimize the cow's fear of the test procedure and to decrease the frequency of responses to touch.

For both the algometer and von Frey devices, the MNT was significantly lower at the dorsal than the lateral stimulation site. This difference might be due to variation in the underlying soft tissue between the two sites (Love et al. 2011). In cattle, a larger amount of soft tissue can be found beneath the lateral stimulation site compared to the dorsal site, thus dissipating the stimulus force and resulting in the higher MNT values. Another possible explanation could be a difference in the density of nociceptors on the two sites (unknown in cattle) as high density of nociceptors has been correlated with lowered thresholds in humans (Selim et al. 2010). Our results thus show that for comparisons of the MNT a small stimulation site should be chosen.

We used two probes with very different configurations. Comparison of MNT just by applied pressure (pressure = force/area) may therefore be inadequate. However, given mean threshold values around 4.2 kgf and 400 gf for the algometer and von Frey respectively, the calculated pressures applied per probe area would be approximately 1242 and 13,075 kilo Pascal (kPa). Pressure from larger probes, as the algometer used, may activate larger amount of nociceptors (spatial summation) resulting in lower thresholds compared with smaller probes (Nie et al. 2009). Further, smaller probes create larger deformation (relative to the probe size) of tissue than larger probes (Treede et al. 2002; Taylor & Dixon 2012a). Hence, the high pressure threshold of the von Frey device may have been influenced by a spread of force over a larger (relative to the probe size) area in the tissue.

The adverse mechanical effects related to the probe configuration could have biased the results. The tip of the von Frey device tended to bend when exposed to maximum force. This could have overestimated the thresholds as the proportion of measured force used to bend the tip was projected sideways and therefore not applied to the skin. For the algometer, where the application of the maximum force could be relatively physical challenging for the observers, the rate of application could have decreased as the stimulus reached the high end of the force range. As lower rates are related to lower threshold values (Jensen et al. 1986), this could have decreased the thresholds in our study. Together with the relatively high frequency of safety end points, the algometer with a 6.5 mm diameter probe might not be appropriate in future studies investigating hyperalgesia on the metatarsus of dairy cattle.

One reason for choosing handheld methodology to be used in the home environment of the loose-housed animals was to evaluate a procedure without restraining the cows other than by the presence of observers. However, not all experimental cows were found in a cubicle on all experimental days. Furthermore, on average 37% and 45% of the cows included in the two experiments ‘dropped out’ of the test before completion. This large number of ‘drop-outs’ would be problematic in future analyses of effects of claw lesions on MNT, especially if cows that could not be tested were confounded with a special range of threshold values, for example if cows with the highest level of fear of the test procedure tended to leave the cubicles earlier and also tended to have lower thresholds. Even if bias did not occur, the high degree of drop-outs resulted in loss of statistical power (Myers 2000). Therefore, in future research, action needs to be taken to reduce the number of drop-outs.

## Conclusion

The present study is among the first to use handheld methodology to quantify MNT in loose-housed dairy cows. The handheld methods have to be further developed to address 1) the relatively high CV, 2) the poor inter-observer agreement for the von Frey device, 3) the low test-retest reliability over time and 4) the high number of drop- outs. We suggest further studies focusing on habituation of cows to the test procedure, including a low stress restraining procedure and using stimulation devices, where the rate of loading force can be controlled, in order to improve the reliability of handheld MNT procedures.
